# Measuring Cytotoxicity by Bioluminescence Imaging Outperforms the Standard Chromium-51 Release Assay

**DOI:** 10.1371/journal.pone.0089357

**Published:** 2014-02-19

**Authors:** Mobin A. Karimi, Eric Lee, Michael H. Bachmann, Ana Maria Salicioni, Edward M. Behrens, Taku Kambayashi, Cynthia L. Baldwin

**Affiliations:** 1 Department of Veterinary & Animal Sciences, University of Massachusetts, Amherst, Massachusetts, United States of America; 2 Department of Pathology and Laboratory Medicine, Perelman School of Medicine at the University of Pennsylvania, Philadelphia, Pennsylvania, United States of America; 3 Department of Pediatrics, Stanford University School of Medicine, Stanford, California, United States of America; 4 Department of Pediatrics, The Children’s Hospital of Philadelphia, Philadelphia, Pennsylvania, United States of America; ENEA, Italy

## Abstract

The chromium-release assay developed in 1968 is still the most commonly used method to measure cytotoxicity by T cells and by natural killer cells. Target cells are loaded *in vitro* with radioactive chromium and lysis is determined by measuring chromium in the supernatant released by dying cells. Since then, alternative methods have been developed using different markers of target cell viability that do not involve radioactivity. Here, we compared and contrasted a bioluminescence (BLI)-based cytotoxicity assay to the standard radioactive chromium-release assay using an identical set of effector cells and tumor target cells. For this, we stably transduced several human and murine tumor cell lines to express luciferase. When co-cultured with cytotoxic effector cells, highly reproducible decreases in BLI were seen in an effector to target cell dose-dependent manner. When compared to results obtained from the chromium release assay, the performance of the BLI-based assay was superior, because of its robustness, increased signal-to-noise ratio, and faster kinetics. The reduced/delayed detection of cytotoxicity by the chromium release method was attributable to the association of chromium with structural components of the cell, which are released quickly by detergent solubilization but not by hypotonic lysis. We conclude that the (BLI)-based measurement of cytotoxicity offers a superior non-radioactive alternative to the chromium-release assay that is more robust and quicker to perform.

## Introduction

Cellular cytotoxicity plays an important role in the immune response [1]. There are two main types of cytotoxic cells: natural killer (NK) cells and cytotoxic T lymphocytes (CTLs). NK cells interact with other cells via an inhibitory receptor and/or an activating receptor, while CTLs utilize the T-cell receptor to recognize antigenic peptides bound to major histocompatibility complex molecules [2]. Both induce target cell lysis through perforin and granzyme release [3], while CTLs can also induce apoptosis through Fas-Fas ligand interactions [4].

To measure the cytotoxic activity of effector cells *in vitro*, the chromium release cytotoxicity assay has widely been used [2], [5], [6]. The chromium release assay is still the gold standard in measuring CTL or NK cell-mediated cytotoxicity, especially when using primary cell targets. However, the chromium release assay is limited to a single time point readout only, as the same sample cannot be measured at more than one time point. Many institutions encourage researchers to limit the time spent in the use of the exposure to radioactive materials. As ^51^Cr is radioactive, it is harmful to the researcher’s health and it requires special radioactive training. Moreover, the cost of safe disposal of radioactive waste must be considered and measuring ^51^Cr requires a gamma counter, which is an expensive instrument. Thus, safer alternative methods to measure the cytotoxicity of cells are urgently needed.

A number of alternative methods have been developed that avoid the use of radioactive reagents. Non-radioactive chromium can be used to label target cells and its release from lysed cells can be measured by flameless atomic absorption spectroscopy [7]. Correlation of data from this method and the radioactive chromium-release assay is high [1]. However, FAAS takes a long time to measure chromium-release, as a single sample takes up to 2 minutes to read, making the processing of large numbers of samples problematic. As an alternative approach to chromium release, flow cytometric assays that use fluorescent dyes such as carboxyfluorescein succinimidyl ester (CFSE), PKH-2, and PKH-26, which are lipophilic and integrate into the cell membrane, have been developed [8]–[10]. These dyes have been used for multiple color analysis via flow cytometry to measure cytotoxicity by human and mouse effector cells [10]. Comparisons between these flow cytometric methods and the chromium-release assay have shown that the flow cytometric methods generated similar or even higher lysis, suggesting that flow cytometric methods could be more sensitive than the chromium-release assay. Another advantage of using fluorescent dyes is that co-staining with propidium iodide or with Annexin V clearly distinguishes live and dead cells, while a second set of reagents labeled with different dyes and fluorescent antibodies allows distinction between effector and different populations of target cells by flow cytometry [8].

Another approach utilizes bioluminescence (BLI) of luciferase-transduced cell lines. BLI imaging is based on the detection of light from a variety of light-emitting enzymes such as luciferases using highly sensitive cameras or luminometers [11]. Luciferase is used most frequently for BLI imaging and has been engineered in several iterations for improved performance in mammalian cells [11]. Since BLI is ATP-dependent, a dying cell will stop emitting BLI once its remaining intracellular ATP has been used up. Thus, by stably transducing tumor target cell lines to express luciferase, NK and CTL cellular cytotoxicity can be detected as a decrease in BLI. BLI was first used by Brown et al. and subsequently by others [12]–[15] to rapidly measure the cytotoxic activity of effector cells. BLI-based cytotoxicity assays are also referred to as compartment-specific bioluminescence imaging (CS-BLI), since the specific lysis of tumor targets can be detected even in the presence of accessory cell types such as stromal cells, which can be useful in high throughput drug screening [12]–[14].

In this report, we performed an extensive analysis comparing the utility of the BLI-based assay and the chromium release assay using several human and murine target cell lines [2]. Our data demonstrate that the BLI-based assay is more robust, exhibits an increased signal-to-noise ratio, and displays faster kinetics. In all our cytotoxivity assays were performed on the same time, and the same cell line. The reduced cytotoxicity detected by the chromium release method was likely due to the association of chromium with structural components of the cell, which cannot be released upon target cell lysis. We conclude that the BLI-based measurement of cytotoxicity offers a superior non-radioactive alternative to the chromium release assay that is more robust and quicker to perform.

## Materials and Methods

### 2.1 Ethics Statement

For human samples, all volunteered participated in this study were provided informed consent form. All participants provided their written consent form before participating in this study. The consent form and procedure were approved by the ethics committees according to the according to the Institutional Review Board at the University of Massachusetts (IRB) protocol. Protocol ID: 2011–1051, were approved by Institutional Review Board at the University of Massachusetts (IRB) The Institutional animal care and use committee at the University of Massachusetts approved all mouse experiments.

### 2.2 Generation of Luciferase-expressing Cell Lines

The cell lines K-562 (human chronic myelogenous leukemia (CML); ATCC CLL-243), U266 (human myeloma plasmacytoma; ATCC TIB-196), EL-4 T cell lymphoma (C57BL/6N; ATCC-TB-39), P815 (Mastocytoma mast cell line; ATCC TIB-64), and YAC1 (lymphoma moloney murine leukemia virus (Mo-MuLV)-induced cell line; ATCC-TIB-160) were purchased from ATCC. UCI101 was a kind gift from Dr. John Chen from Stanford University [15]. All the tumor cell lines were transduced with luciferase as described [15] and used as targets in cytotoxicity assays. The luciferase-expressing A20 cell line was a kind gift of Dr. Negrin (Stanford University), which was lentivirally transduced with a puromycin resistance gene. Stable retroviral transduction of tumor cell lines was performed using a modified pQCX-I-GLF vector containing firefly luciferase fused to GFP, which was subcloned from the pHR2-GLF vector (kindly provided by Dr. R.J. Creusot, Stanford University, CA) [16]. To produce retroviral supernatants, Phoenix packaging cells were plated in 175 cm^2^ flasks, and transfected with 20 µg of vector using Lipofectamine 2000 reagent (Invitrogen, Carlsbad, CA), according to the manufacturer’s protocol. To produce lentiviral supernatants, 293T cells were plated in 175 cm^2^ flasks, and transfected with 10 µg of plenti vector, 8 µg Gag/Pol, and 2 µg of VSV-G using Lipofectamine 2000 reagent as stated above. The medium was changed after 8–12 hours, and viral supernatants were harvested after 24–36 hours, and ultracentrifuged for 140 min at 19,500 rpm at 4°C. Concentrated viral supernatants were re-suspended in IMDM media (Invitrogen) and used to transduce human and murine target cell lines in the presence of polybrene (10 µg/ml) and protamine sulfate (10 µg/ml) to enhance transduction efficiency. Stable viral transductants were sorted twice for GFP fluorescence using a FACS DIVA cell sorter. Sorted cells were expanded and screened for bioluminescence using a Xenogen IVIS Spectrum (Caliper Life Sciences; Hopkinton, MA).

### 2.3 Generation of Effector Cells

Peripheral blood lymphocytes from healthy human donors or murine splenocytes were cultured for 1–3 weeks and activated and expanded as previously described [3], [17]. Briefly, mononuclear cells were isolated from healthy donors by Ficoll-Hypaque density centrifugation. The final product was re-suspended at 2 × 10^6^ cells/mL in complete RPMI consisting of 10% FCS, 2 mM L-glutamine, 100 U/mL penicillin, 100 µg/mL streptomycin, and 50 µg/mL 2-mercaptoethanol at 37°C. On day 0, cells were activated with IFN-γ (1000 U/mL; Genentech, South San Francisco, CA) and the following day stimulated with anti-CD3 (OKT-3 at 25 ng/mL; OrthoBioTech, Raritan, NJ) and recombinant IL-2 (rIL-2; 300 U/mL; Chiron, Emeryville, CA). Thereafter, rIL-2 (300 U/mL) was added every 3 to 5 days and cells maintained at a density of 1.5 to 2 × 10^6^/mL for a total of 14–28 days. To obtain mouse effector cells, murine spleen cells were stimulated with plate-immobilized anti-CD3 mAb (25 ng/mL) for a day, after which IFN-γ (1000 U/mL) and recombinant IL-2 (rIL-2; 300 U/mL) were added. Thereafter, rIL-2 (300 U/mL) was added every 3 to 5 days and cells were maintained at a density of 1.5 to 2 × 10^6^/mL for a total of 14–28 days. For human samples, consent was obtained from all donors of peripheral blood lymphocyte buffy coats under protocols approved by the Institutional Review Board at the University of Massachusetts. Mouse experiments were approved by the Institutional Animal Care and Use Committee at the University of Massachusetts.

### 2.4 BLI-based Cytotoxicity Assay

Luciferase-expressing tumor cells were placed in 96–well round bottom plates at a concentration of 3×10^5^ cells/ml in triplicates, were given D-firefly luciferin potassium salt (75 µg/ml; Caliper Hopkinton, MA), and measured with a luminometer. This was done to establish the BLI baseline readings before the occurrence of any cell death and to ensure equal distribution of target cells among wells. Subsequently, effector cells were added at 40∶1, 20∶1, 10∶1, and 5∶1 effector-to-target (E:T) ratios and incubated at 37°C for 2 or 4 hours. BLI was then measured for 10 seconds with a luminometer (Packard Fusion Universal Microplate Analyzer, Model A153600) as relative light units (RLU). Cells were treated with 1% Nonidet P-40 (NP40) or with water as a measure of maximal killing. Target cells incubated without effector cells were used to measure spontaneous death RLU. Cells were images at 2 hours or 4 hours. Triplicate wells were averaged and percent lysis was calculated from the data with the following equation: % specific lysis = 100×(spontaneous death RLU – test RLU)/(spontaneous death RLU – maximal killing RLU).

### 2.5 Chromium-release Assay

The luciferase-transduced tumor cell lines were labeled with ^51^Cr by incubating 1×10^6^ cells in 300 µCi of ^51^Cr for 2 hours at 37°C. Labeled cells were washed 3 times with PBS or re-suspended in complete RPMI medium, and plated in 96-well round bottom plates in triplicates at a concentration of 1×10^5^ cells/ml. Effector cells were added in 40∶1, 20∶1 10∶1 and 5∶1 effector-to-target ratios and plates incubated at 37°C for 2 or 4 hours. Supernatants were collected and the released ^51^Cr was counted using a gamma counter. Cells were treated with 1% NP40 or with water as a measure of maximal killing. Target cells incubated without effector cells were used to measure spontaneous ^51^Cr release. Counts from triplicate wells were averaged and then percent lysis was calculated using the following equation: % specific lysis = 100×((test ^51^Cr release) – (spontaneous ^51^Cr release))/((maximal ^51^Cr release)−(spontaneous ^51^Cr release)).

### 2.6 Statistical Analysis

Statistical significant differences between the measured cytotoxicity of Chromium release versus BLI assays over varying effector to target ratios were determined by permutated two-way ANOVA using the method of Manly [18] in order to avoid assumptions regarding the normality of the distribution of the data. 5000 permuations were used for each test. An R script was written to perform the test borrowing code from the following website: http://www.uvm.edu/~dhowell/StatPages/More_Stuff/Permutation%20Anova/PermTestsAnova.html. P-values of less than 0.05 were considered statistically significant.

## Results

### 3.1 The BLI-based Cytotoxicity Assay Exhibits a Higher Signal to Noise Ratio and is More Robust Compared to the Chromium-release Cytotoxicity Assay

Cytotoxicity assays are designed to measure the percentage of cells that are killed by effector cells over a defined period of co-culture. Typically, the assay is carried out over 4 hours at increasing E:T ratios. The % cytotoxicity is then calculated by measuring target cell viability. Target cell viability is determined by the amount of radioactive chromium released into the supernatant (chromium release assay) or by measuring the luciferase activity in the surviving target cells (BLI method). Because there could be considerable background (spontaneous death or spontaneous release/reduction in signal) associated with these measurements, it is critical that a reasonable signal to noise (maximum to minimum) ratio is achieved for precise measurements. To compare the signal to noise ratio of the chromium release assay to the BLI-based assay, we labeled a variety of luciferase-transduced human and mouse cell lines with ^51^Cr. The labeled cells were cultured in media or lysed with 1% NP40 for 4 hours and the maximal death (1% NP40 lysis) and background values (cultured in media alone) were obtained. Compared to the chromium release assay, the BLI assay displayed a significantly higher signal to noise ratio in all but one of the cell lines (Table 1). The maximum/minimum values and the dynamic range of both assays are shown in Table 1.

**Table 1:** Assay characteristics of the chromium release assay vs. BLI assay.

	Chromium Release				BLI			
Cell line	Spontaneous	Max	Range	Max:Min ratio	Spontaneous	Max	Range	Max:Min ratio
K562	3716±728	21862±473	18146	5.9±3.4	77.9±2.1	24.6±0.20	53.3	3.2±3.2
U266	1669±2 6	24303±801	22633	14.6±4.1	499.3±11.0	23.0±1.0	476.3	21.7±2.1
UCI101	1296±349	11624±149	10328	9.0±3.1	372.3±7.1	23.3±1.5	349	16.0±3.2*
P815	2610±12	14669±299	12058	5.6±3.1	472.3±7.1	24.7±0.6	447.6	19.1±4.1*
YAC1	517±193	6945±484	6428	13.4±32	653.3±5.8	23.6±0.9	629.7	27.7±2.1*
EL-4	906±28	4561±149	3655	5.0±2.1	672.3±7.1	23.7±1.5	648.6	28.4±3.1*
A20	3480±115	14741±612	11261	4.2±3.5	259.0±33.8	23.6±0.9	235.4	11.0±2.1*

The spontaneous death, maximal killing, range, and the signal to noise (max:min) ratios measured by ^51^Cr release and BLI are shown. Results are presented as mean ± SD of three independent experiments.

* p<0.01 by Student t test.

**Figure 1:**
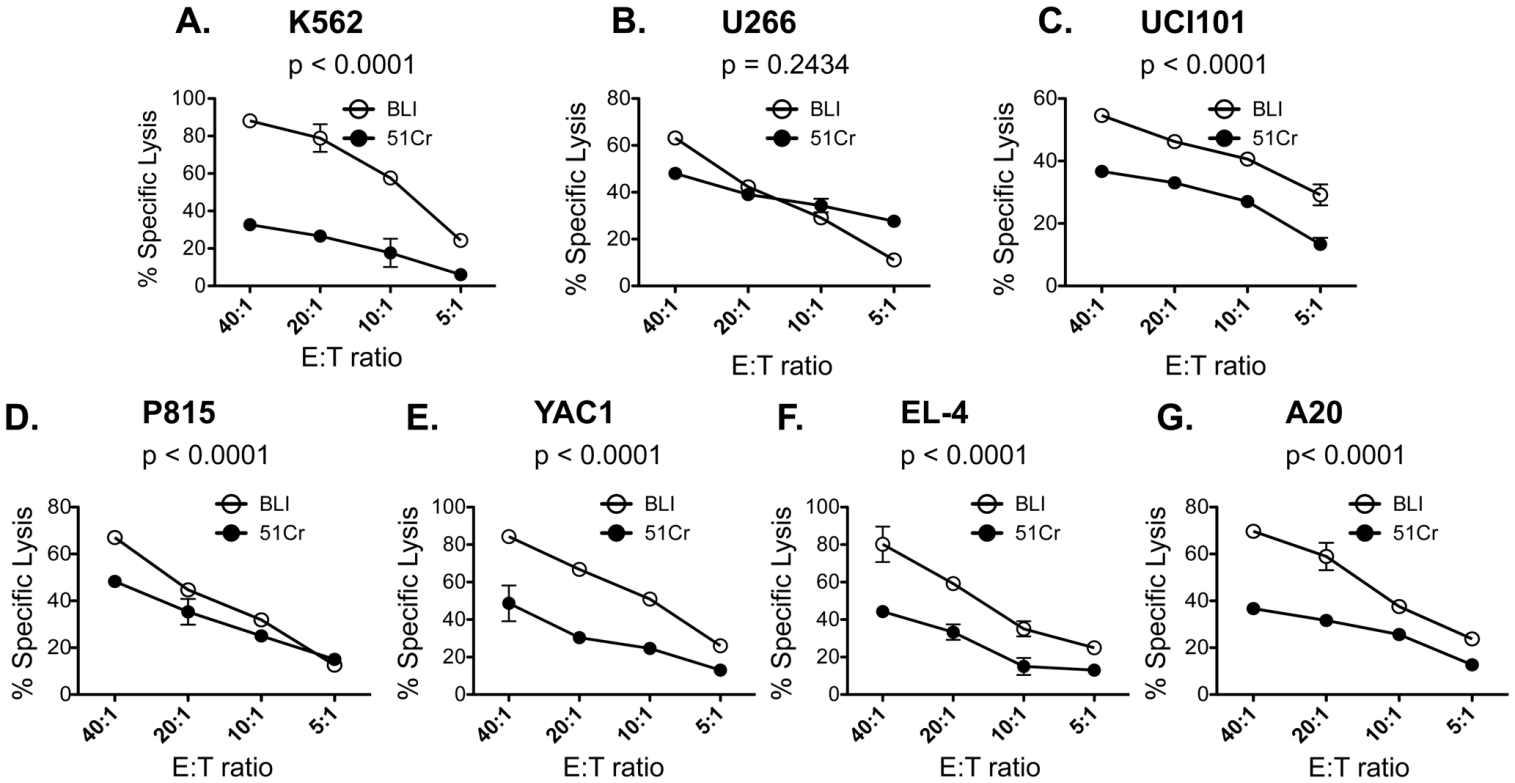
Comparison of cytotoxicity obtained at 4 hours by the chromium release and BLI method. Luciferase-transduced human and mouse cell lines were co-cultured with human or mouse effector cells for 4 hours at various E: T ratios. The % specific lysis of the human cell lines, (A) K562, (B) U266, and (C) UCI101 obtained by the chromium release assay (closed circles) or the BLI assay (open circles) is plotted against multiple E:T ratios. The % specific lysis of the murine cell lines, (D) P815, (E) YAC1, (F) EL-4, and (G) A20 obtained by the chromium release assay (closed circles) or the BLI assay (open circles) is plotted against multiple E:T ratios. Results are represented as mean ± SD of n = 3 independent experiments. The p values obtained from the statistical analysis performed by permutated two-way ANOVA are shown in each graph.

**Figure 2:**
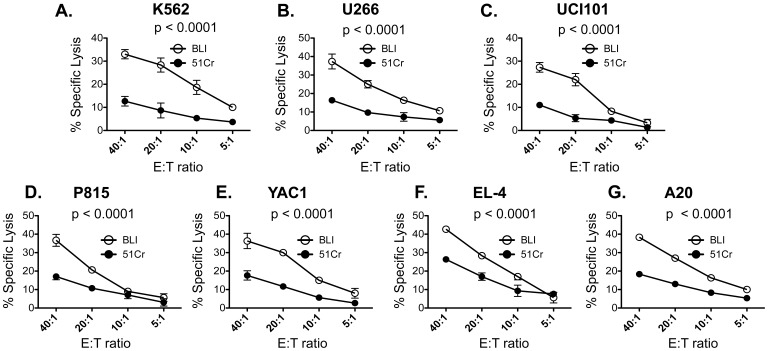
Comparison of cytotoxicity obtained at 2 hours by the chromium release method and BLI method. Luciferase-transduced human and mouse cell lines were co-cultured with human or mouse effector cells for 2 hours at various E:T ratios. The % specific lysis of the human cell lines, (A) K562, (B) U266, and (C) UCI101 obtained by the chromium release assay (closed circles) or the BLI assay (open circles) is plotted against multiple E:T ratios. The % specific lysis of the human cell lines, (D) P815, (E) YAC1, (F) EL-4, and (G) A20 obtained by the chromium release assay (closed circles) or the BLI assay (open circles) is plotted against multiple E:T ratios. Results are represented as mean ± SD of n = 3 independent experiments. The p values obtained from the statistical analysis performed by permutated two-way ANOVA are shown in each graph.

**Figure 3:**
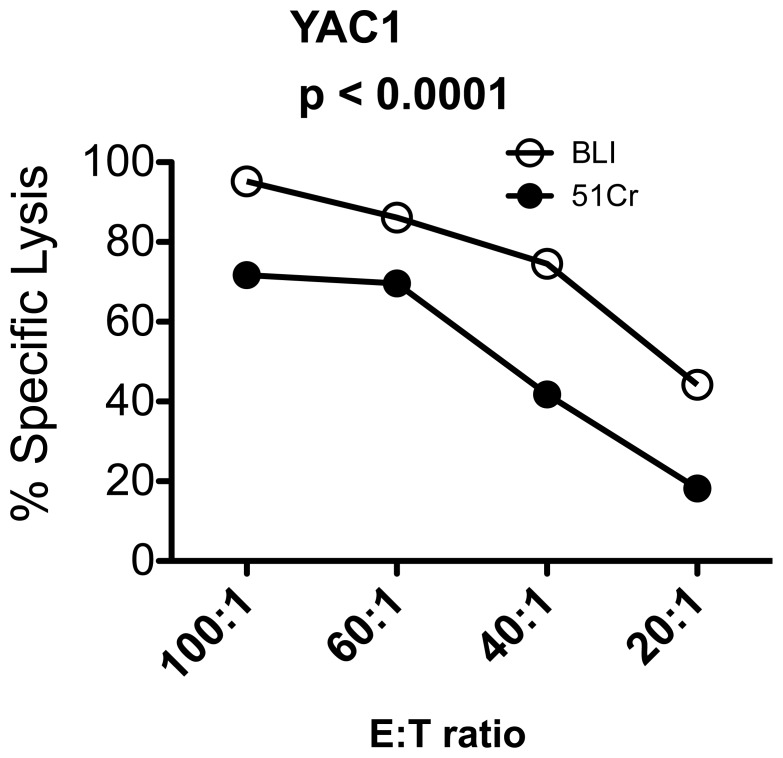
Comparison of cytotoxicity obtained at 4 hours by the chromium release and BLI method at higher E:T ratios. Luciferase-transduced the YAC1 cell line was co-cultured with mouse effector cells for 4 hours at various E:T ratios ranging from 100∶1 to 20∶1. The % specific lysis obtained by the chromium release assay (closed circles) or the BLI assay (open circles) is plotted against multiple E:T ratios. Results are represented as mean ± SD of triplicate values. One representative of 2 independent experiments is shown. The p value obtained from the statistical analysis performed permutated two-way ANOVA is shown.

Next, to compare the performance of the chromium-release assay to the BLI-based assay, we co-cultured a variety of ^51^Cr-labeled luciferase-transduced human and mouse cell lines with cytotoxic effector cells for 2 or 4 hours. As shown in Figure 1 and 2, the degree of cytotoxicity was directly proportional to the E:T ratio for both assays**.** The same target cell line, were used in both BLI and the chromium-release assay. However, with the exception of U266, the % specific lysis obtained by the BLI method at 4 hours was higher for all cell lines tested with an average increase of 186% ±53% (mean ± SD of all cell lines) at the 40∶1 E: T ratio (Fig. 1). This difference was even more pronounced when the co-culture was shortened to 2 hours with an average increase of 245% ±30% (mean ± SD of all cell lines) at the 40∶1 E: T ratio (Fig. 2). At 2 hours, a statistically significant difference in % lysis between the BLI and the chromium-release assay was observed even with the U266 cell line. Moreover, the % lysis obtained at 2 hours by the BLI method was comparable to the % lysis obtained at 4 hours by the chromium release assay. To ensure that the 40∶1 E:T ratio was at a sufficiently high E:T ratio to compare cytotoxicity between the two assays, we performed cytotoxicity assays with one of the cell lines (YAC1) at a 100∶1, 60∶1, 40∶1 and 20∶1 E:T ratio. Although an increase in cytotoxicity was seen when the E:T ratio was increased from 40∶1 to 60∶1, no further increases were seen at the 100∶1 ratio (Fig. 3). However, even at the 100∶1 E: T ratio, the BLI-based assay exhibited a significantly higher cytotoxicity value compared to the chromium release assay. Thus, the BLI-based cytotoxicity assay appears to yield a more robust readout of cytotoxicity.

### 3.2 Differences in Detergent-mediated and Hypotonic Lysis Potentially Explain Why the Chromium Release Assay Underestimates the Degree of Cytotoxicity

**Figure 4:**
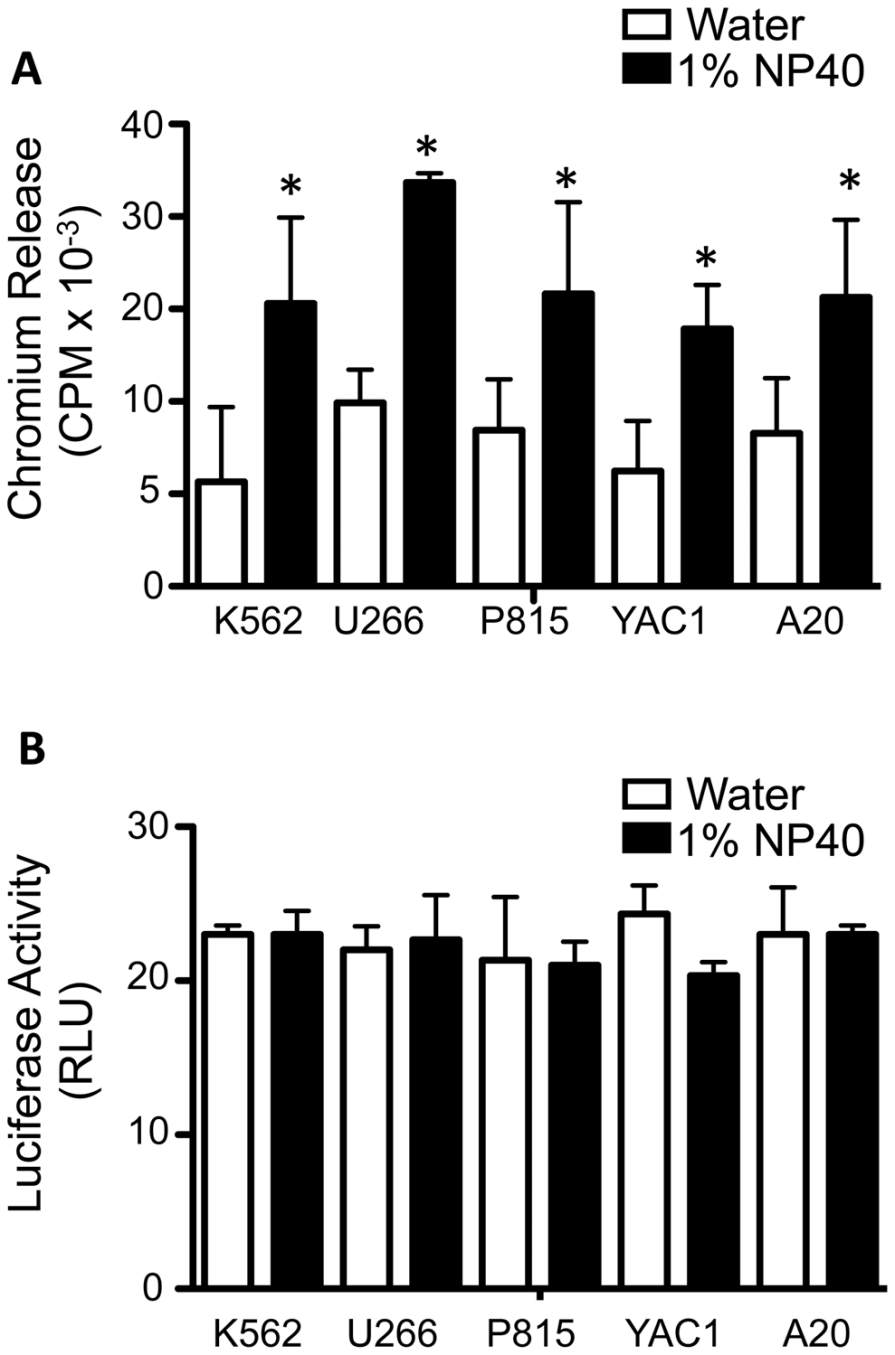
Measurement of chromium release and luciferase activity in cells lysed by water or by 1% NP40. Three luciferase-transduced human cell lines (K562, UCI191 and U266) and 3 mouse cell lines (P815, YAC1, and A20) were labeled with radioactive chromium for 4 hours. The cells were lysed in water or 1% NP40 for 4 hours. (A) Chromium release in the supernatant of cells lysed in water or in 1% NP40 was determined. (B) Luciferase activity was detected by BLI in cells lysed in water or in 1% NP40 was determined. The results are represented as mean ± SD of n = 3–4 independent experiments. * p<0.001 by Wilcoxon Rank test.

As described above, the BLI assay resulted in ∼2-fold more cytotoxicity than observed with the chromium release assay. We hypothesized that these differences could arise because of the highly adhesive and non-specific nature of chromium binding to cell membranes [19]–[21]. Therefore, lysis of cells by effector cells may induce release of cytoplasmic but not cell membrane-associated chromium, leading to apparently lower cytotoxicity values. To test this hypothesis, we labeled three human and three murine cell lines with ^51^Cr and lysed the cells with NP40 or with water. We reasoned that compared to detergent (NP40), which would solubilize the membrane, hypotonic lysis by water would more closely mimic lysis by cells. Indeed, cells hypotonically lysed with water showed significantly less ^51^Cr release compared to the same cells lysed by NP40 (Fig. 4A–B). In contrast, lysis by either water or detergent led to equivalent measurements by the BLI method. Thus, we propose that the reduced detection of cytotoxicity by the chromium release method is attributable to the association of chromium with structural components of the cell, which are released quickly by detergent solubilization but not by hypotonic lysis (Fig. 4A–B).

## Discussion

Cytotoxicity of target cells is a major function exhibited by CTLs and NK cells. To measure the cytotoxic activity of effector cells *in vitro*, the chromium-release cytotoxicity assay has widely been used [2], [5], [6] and has been the gold standard for measuring cytotoxicity of primary tumor samples [6]. However, one major concern with using ^51^Cr is that, unshielded, 1 mCi of ^51^Cr generates as much as 180 millirem per hour of x-rays and gamma radiation, and thus requires more lead shielding than other isotopes in biomedical research. Moreover, ^51^Cr release by target cells can be measured only at a single time point, since the same cell supernatants cannot be measured at multiple time points.

Due to the above limitations with the chromium release assay, several alternative methods that do not use radioactivity have been developed. Dyes such as CFSE, PKH-2, and PKH-26 that are commonly used to detect cell proliferation have been successfully used to detect cytotoxicity against tumor cells lines and virally infected PBMCs by flow cytometry. Detection of cell death by 7AAD and Annexing V staining are other widely used flow cytometric methods to measure CTL cytotoxicity of target cells.

The use of BLI to measure target cell death is a relatively newer method to detect cytotoxicity. BLI involves the use of luminometers to detect bioluminescence emitted from substrates cleaved by luciferases [11]. Because luciferase activity is ATP-dependent, dying cells will stop producing bioluminescence once its remaining intracellular ATP has been used up. Thus, by stably transducing tumor target cell lines to express luciferase, NK and CTL cellular cytotoxicity can be detected as a decrease in bioluminescence. Indeed, a number of previous studies [12]–[15] have shown that the BLI-based cytotoxicity assay can be used to measure the cytotoxic activity of effector cells.

The aim of our present study was to compare the performance of the BLI-based cytotoxicity and the chromium release cytotoxicity assay. Using several luciferase-expressing tumor cells, we measured cytotoxicity by effector cells using the BLI method in parallel with the chromium release assay. Our results demonstrate that the BLI method has many advantages over the chromium release assay. First, the BLI method avoids the use of radioactive materials and is therefore considerably safer. Second, the BLI-based method is quicker and easier to perform than the chromium-release assay. The chromium release assay requires 2-hour labeling time of target cells, while measuring BLI can be performed immediately after the addition of the luciferase substrate D-luciferin. In addition, harvesting and measurement of each sample using the chromium-release assay is cumbersome and can only be performed at one time point for a given sample. This is because the chromium release assay requires the plate to be centrifuged and supernatants to be transferred into tubes for radioactivity measurement. In contrast, since the supernatant of the cells does not need to be removed in the BLI assay, bioluminescence of the whole plate can be quantified quickly and at multiple time points with a luminometer or IVIS system. Thus, the BLI assay is rapid and technically simple to perform. The simplicity of the BLI assay also diminishes the likelihood of technical errors as it reduces sample handling for its set-up and measurements. Third, the BLI method provides an increased signal-to-noise ratio compared to the chromium release assay. An analysis of the ratio between the maximum release (full lysis) and the spontaneous release (background) demonstrated that the BLI method was superior over the chromium release assay. A higher signal:noise ratio will introduce less error in the calculations of cytotoxicity. In addition, the ability to get readings at multiple time points further reduces errors. Together, the simplicity of the assay and the larger signal-to-noise ratio would translate into more precise measurements of cytotoxicity by the BLI method compared to the chromium release assay.

Finally, the BLI assay appears to be more robust than the chromium release assay. Although a similar cytotoxicity trend was observed over multiple E: T ratios with the chromium-release assay and the BLI assay, the BLI assay exhibited significantly larger degrees of cytotoxicity. For example, in cell lines where 70% percent specific lysis was obtained at the 40∶1 E:T using the BLI method, the corresponding value for the chromium-release assay was about half of that at ∼35%. This was consistently observed across almost all human and mouse cell lines tested. We speculated that this difference could be a result of the highly adhesive and non-specific nature of chromium binding to cell membranes [19]–[22]. Indeed, we found that the chromium released by cells treated with detergent solubilization was dramatically higher than when the cells were hypotonically lysed. It is possible that cell membrane-associated chromium takes time to be released and that using detergent solubilization to obtain the maximal release value is an overestimation, leading to falsely low relative cytotoxicity values. Although using hypotonic lysis values as a maximum may correct the underestimation of the cytotoxicity, this would result in a further reduction in the signal-to-noise ratio in the chromium release assay. In contrast, as the BLI assay relies on decreased luciferase activity by depletion of intracellular ATP, the localization of luciferase becomes irrelevant. This is reflected in our data demonstrating that the BLI values obtained with detergent-mediated and hypotonic lysis were equivalent.

The BLI assay is, however, not without its disadvantages, the major one being that only cells that express luciferase can be used. Primary tumor samples or virus-infected targets, for example, would have to be transduced prior to the assay. In a previous study, Brown et al. [23] used U251T cells or human autologous PBMC target cells loaded with the influenza A matrix coat protein MP1 and transfected them to transiently express luciferase [23]. They showed that cytotoxic activities of a MP1-specific CTL clone against these target cells transiently expressing luciferase was detectable and comparable to stably transduced target cells expressing luciferase [23]. However, transfection takes time, is potentially toxic to some cells, and is available for only limited primary cell types. Therefore, while the BLI method carries more advantages over the chromium release assay when using target cells genetically engineered to express luciferase, the chromium release assay or flow cytometric methods currently remain the methods of choice when measuring cytotoxicity against primary cells.

## Conclusions

In summary, we have compared the performance of the BLI method and the chromium release assay to measure cytotoxicity of many human and mouse cell lines. This was an important study to perform, because BLI-based assays are becoming more widely used in various settings including *in vitro* cytotoxicity assays as well as *in vivo* monitoring of anti-tumor responses. We conclude that the BLI assay is a superior alternative method for measuring cytotoxicity of transfectable cell lines, as the BLI assay is safer, faster, easier to perform, and is more robust compared to the chromium release assay.
